# Supplementary material for: Perceived change in physical activity levels and mental health during COVID-19: Findings among adult twin pairs
Source: PLoS One. 2020 Aug 13;15(8):e0237695. doi: 10.1371/journal.pone.0237695 (PMC7425865; doi:10.1371/journal.pone.0237695)
Supplement: S1 File — (DOCX) [file pone.0237695.s001.docx]

**S1 Text.** Twin Correlations for Exposure and Outcome Variables.

In S1 Table below, we provide information on the twin correlations for perceived stress and anxiety, and tetrachoric twin correlations for perceived change in the amount of physical activity or exercise. The standardized biometric variance components for the three variables are also presented. There was substantial unique environmental variance (E, 60-70%) in the two perceived change in physical activity or exercise comparisons. The additive genetic component (A, 40%) was present in the comparison between those who reported an increase versus those who reported no change in physical activity, whereas the shared environmental contribution (C, 30%) was present in the comparison between those who reported decreased versus those with no change in physical activity. For perceived stress, most of the variance was attributable to the non-shared environmental component (E, 61%), with smaller proportions attributable to additive genetics (A, 23%) and shared environmental factors (C, 16%). For anxiety, 58% of the variance was attributable to the non-shared environmental component (E), with the remaining variance attributable to additive genetics factor (A, 42%).

S1 Table. Twin correlations and standardized variance components for changes in physical activity, perceived stress, and anxiety among same-sex twin pairs.

|  | ***r*MZ** | ***r*DZ** | ***a^2^*** | ***c^2^*** | ***e^2^*** |
| --- | --- | --- | --- | --- | --- |
| **Perceived stress** | **.39 (.03)** | **.27 (.06)** | .23 (.13) | .16 (.12) | **.61 (.03)** |
| **Anxiety** | **.42 (.03)** | **.21 (.02)** | **.42 (.03)** | - | **.58 (.03)** |
| **Change in physical activity or exercise*** |  |  |  |  |  |
| **Increase vs. no change** | **.40 (.09)** | **.20 (.04)** | **.40 (.09)** | - | **.61 (.09)** |
| **Decrease vs. no change** | **.30 (.07)** | **.30 (.07)** | - | **.30 (.07)** | **.70 (.07)** |

Standard errors are presented within parentheses. Estimates that are statistically significant are **bolded**.

*r*MZ*:* monozygotic twin correlations; *r*DZ: dizygotic twin correlations. *a^2^*, *c^2^*, and *e^2^*: standardized biometric variance components obtained from classical twin model decomposing the variance of the phenotype into additive genetic (A), shared environment (C), and non-shared environment (E) variance components, respectively.

*Tetrachoric correlations are presented here due to the dichotomous nature of the change in physical activity comparisons.

**S1 Figure.** Stress and anxiety levels by self-reported change in amount of physical activity or exercise within same-sex twin pairs.

**S2 Figure.** Univariate and quasi-causal twin models.

*A*: additive genetic component; *C*: shared environmental component; *E*: unique environmental component; *b­_A_* and *b­_C_*: amount of residual variance of mental health attributable to the genetic and shared environment, respectively; *b­_phen_*: phenotypic association. Mental health refers to perceived stress or anxiety outcomes, computed in separate models.
