# Supplementary material for: Perceived change in physical activity levels and mental health during COVID-19: Findings among adult twin pairs
Source: PLoS One. 2020 Aug 13;15(8):e0237695. doi: 10.1371/journal.pone.0237695 (PMC7425865; doi:10.1371/journal.pone.0237695)
Supplement: Attachment — Submitted filename: Response to Reviewers.pdf [file pone.0237695.s002.pdf]

PONE-D-20-20044 – Response to Reviewers

Perceived change in physical activity levels and mental health during COVID-19: Findings among adult twin pairs

On behalf of my co-authors, I thank the Academic Editor and the two reviewers for their helpful comments and suggestions.

Below, we provide a point-by-point response to each issue raised. We believe we have addressed all concerns. However, there were a few instances in which we were unclear on what was being asked of us to change. We note those instances below; we ask the Academic Editor to advise us on what further changes should be made in response to those items that were unclear.

Sincerely,

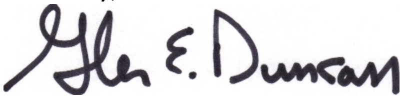

Academic Editor

- "Physical distancing" replaces "social distancing" in the text as suggested.
- A statement regarding lack of causality in observational studies has been added to the limitations section as suggested.

Journal Requirements

- We have made several minor modifications so that the manuscript meets PLOS ONE'S style requirements. We changed file names as well (i.e., simple names referring to the various components).
- The reference to "data available upon request" has been removed (pg. 5, L84); this data is not a core part of the research study being presented.
- The data belong to the Washington State Twin Registry (WSTR). This is an example of a "third-party data" entity that falls under the journals' acceptable data access restrictions. We provide the following details regarding third-party data access directly below.
    - The data supporting the results of the present study are owned by the Washington State Twin Registry (WSTR). Thus, the data cannot be publicly shared as it involves third-party data. However, researchers interested to apply to gain access to the data can do so by contacting the WSTR and completing the appropriate forms stipulated in the WSTR Policies & Procedures guidelines. Application information can be sent to the Scientific Operations Manager at the following URL (https://wstwinregistry.org/contact-us/) or via email (ws.twinregistry@wsu.edu).
- We have updated the Supporting Information files and in-text citations to match accordingly.

Reviewer 1

- Abstract
    - L20-21: We re-wrote this sentence to clarify the terms "outcomes" and "impact".
    - L36-38: We are unclear on the reviewer's comment. The statement in L36-38 (conclusion) maps to L31-35 (results).
    - General: The reviewer recommends adding regression results, names of measures, and statistical designs in the abstract. However, the abstract in its current form is already at

298 words and the limit is 300 words. Please advise on whether these requested changes can be made in the context of the abstract word limit.

- Introduction
  - United States is spelled out at first mention and abbreviated U.S. thereafter.
  - L43: “Has” changed to “have”.
  - L62: The statement “an important strength” was removed from the introduction.
  - L64: We are unclear on the reviewer’s request regarding explaining the role of twin data in the study. In L64-72, we briefly discuss how twin designs allow for the control of genetic and shared environmental confounds in the association between perceived changes in PA levels and mental health, and provide a reference. We believe this is an appropriate level of detail for the introduction that explains why we are using twins (i.e., control for genetic and shared environmental factors that are associated with both the exposure and the outcomes, L66-72 in original paper). Then, we explain the twin approach in detail in the Statistical Analysis subsection of Materials and Methods, including the requested information on the ACE model and the between- and within-pair approach, as well as illustrations of the twin approach in the Supplementary figures (S1_Fig and S2_Fig). Please advise on what further changes are warranted.
  - General: The reviewer queries on existing findings from other studies. We have included information on general findings from 5 studies that we have found, some published within the past few days, related to PA and COVID. Most of what we found on this topic came from reviews, commentaries, and editorials rather than empirical studies.
- Materials and Methods
  - L77: We do not have a reference to the survey we created for this specific study. However, the exact wording of the exposure and outcome variables is provided in the Measures subsection. We could provide the full survey as Supplementary Information if desired. Please advise.
  - L109: “Including the day the participant completed the survey” was added as suggested.
  - L117: “The day the participant completed the survey” was added.
  - L125: The Supplementary Information Figure 1 (S1_Fig) shows non-linear associations between perceived change in PA and both stress and anxiety. The relationship between the exposure and each outcome is “U-shaped”.
  - L128: We clarified this statement as suggested.
  - L131: We included model numbers in the text (see Statistical Analysis section) to map to the results tables.
  - L141: We are unclear on the reviewer’s request regarding the subscripts. The sentence currently reads: The models were then re-estimated including estimates of b_A and/or b_C, which controls for genetic and shared environmental confounds, respectively, in the estimation of the phenotypic association (S2 Figure). Thus, the b_A and b_C components are defined as genetic and shared environmental confounds, respectively. Please advise on necessary changes.
  - L150: We limited the analyses to same-sex twin pairs. First, including opposite-sex twin pairs is complicated analytically. If we include opposite sex twin pairs, then we need to consider sex differences in the models. However, fewer men responded than women, so there was not enough statistical power to perform a 5-group analysis (MM MZ, MM DZ, FF MZ, FF DZ, MF DZ). Instead, we only include sex as a covariate rather than a multiple group analysis to control for any sex differences.

- Results
  - We are unclear on the reviewer's comment related to "the significance of between and within-pair comparisons". The phenotypic model is the between-twin model and the quasi-causal model is the within-pair model. We state differences among the various models in Results and Tables 2 and 3, and discuss their meaning in Discussion. We added descriptors in Discussion to identify which associations are between and within-twin (see L271 and 273).
  - We added p-values in the Results.
- Discussion
  - L219: We respectfully disagree with the suggestion to change the later part of this sentence to "change in/increase in stress" because the outcome variables did not query on changes in stress/anxiety but rather stress and anxiety levels in the last two weeks at one point in time. The direction of association provides information on the nature of the relationship, however, we cannot accurately say "change in" within the context of the observational study design.
  - L223: See comment in L219 above.
  - L249: Change made as suggested.
  - L251: We changed "it is likely" to "possible reasons" as suggested.
  - L283: We modified this statement and removed "by country" as suggested.
  - L285: "Also" was added in this sentence to denote moving to a different topic.
  - L287: "In addition" was added to this sentence to denote a new topic.

Reviewer #2

1. Abstract: The abstract is currently 298 words and the limit is 300 words. Please advise on what changes, if any, can be made within the context of the 300-word limit.
2. L40: COVID-19 was spelled out at first appearance as suggested.
3. We did consider depression as an outcome, however, the PHQ-2 scale that we use in our larger WSTR survey addresses depression symptoms rather than depression per se. Overall, we find low levels of depressive symptoms among WSTR twins, with mean scores ~1 out of a possible 6 points. Further, the PHQ-2 scale assesses "global" rather than acute depressive symptoms. For these reasons, we decided to focus on stress and anxiety.
4. Introduction (above L73): We made some changes to tense for consistency.
5. L79: We note the implementation of the WA "stay home, stay healthy" order on March 24, 2020 in the discussion around L274.
6. L98: The sentence beginning with "To address the potential for recollection bias" has been removed as suggested.
7. We include information on reliability of the stress and anxiety scales under the Measures subsection.
8. We are not clear on what the reviewer is requesting regarding basic data of the sample. We provide standard descriptive statistics including means and standard deviations or frequencies. We added reliabilities for the outcomes as noted in item #7. We square-root-transformed the outcomes because they were highly positively skewed (L146-147 of original paper). However, we did not apply a formal test to determine this based on the descriptive plots.
9. To clarify, we divided age by 10 so that variables would be on similar scales (see L147-148 of original paper). However, we did not categorize age by 10.
10. We are unclear about what changes are being requested and to which figures. Please advise.

11. We deleted the statement beginning on L243 of the original paper that refers to "enough power to detect the difference ….". We wrote this statement merely as a speculative discussion point rather than a definitive statement on statistical power.
12. L272: The reference to California was removed.
